# IGF-II transgenic mice display increased aberrant colon crypt multiplicity and tumor volume after 1,2-dimethylhydrazine treatment

**DOI:** 10.1186/1477-3163-5-24

**Published:** 2006-11-21

**Authors:** Daniela Diehl, Doris Oesterle, Martin W Elmlinger, Andreas Hoeflich, Eckhard Wolf, Harald Lahm

**Affiliations:** 1 Institute of Molecular Animal Breeding and Biotechnology, Ludwig-Maximilians University, Feodor-Lynen-Str. 25, D-81377 Munich, Germany; 2 Institute of Toxicology, GSF-National Research Center for Environment and Health, Ingolstädter Landstr.1, D-85764 Neuherberg, Germany; 3 Pediatric Endocrinology, Children's Hospital, University of Tübingen, Hoppe-Seyler-Str.1, D-72076 Tübingen, Germany

## Abstract

In colorectal cancer insulin-like growth factor II (IGF-II) is frequently overexpressed. To evaluate, whether IGF-II affects different stages of tumorigenesis, we induced neoplastic alterations in the colon of wild-type and IGF-II transgenic mice using 1,2-dimethylhydrazine (DMH). Aberrant crypt foci (ACF) served as markers of early lesions in the colonic mucosa, whereas adenomas and carcinomas characterized the endpoints of tumor development. DMH-treatment led initially to significantly more ACF in IGF-II transgenic than in wild-type mice. This increase in ACF was especially prominent for those consisting of ≥three aberrant crypts (AC). Nevertheless, adenomas and adenocarcinomas of the colon, present after 34 weeks in both genetic groups, were not found at different frequency. Tumor volumes, however, were significantly higher in IGF-II transgenic mice and correlated with serum IGF-II levels. Immunohistochemical staining for markers of proliferation and apoptosis revealed increased cell proliferation rates in tumors of IGF-II transgenic mice without significant affection of apoptosis. Increased proliferation was accompanied by elevated localization of β-catenin in the cytosol and cell nuclei and reduced appearance at the inner plasma membrane. In conclusion, we provide evidence that IGF-II, via activation of the β-catenin signaling cascade, promotes growth of ACF and tumors without affecting tumor numbers.

## Background

Aberrant crypt foci (ACF) are generally accepted to represent precursor lesions in stepwise colon cancer development and emerge in the colon of rodents after treatment with 1,2-dimethylhydrazine (DMH) [1,2]. ACF were also found in humans who underwent surgery for colorectal cancer [3]. ACF in an unembedded colon are characterized by increased size of the crypts, a thickened layer of epithelial cells, an increased pericryptal space and an irregular lumen [4,5]. Although in carcinogen-treated rodents and patients with sporadic colorectal cancer (CRC) only a small fraction of ACF progresses to a tumor, there is a clear association between the development of ACF and tumor formation, at least in the colon of rodents [6]. These data are further supported by the observation that patients with colon cancer resident in regions with high incidence rates of colorectal cancer have higher density of ACF in the colonic mucosa than patients from low incidence regions. Moreover, patients with familial adenomatous polyposis (FAP) ACF showed definite dysplasia in 75–100% of the cases [6], whereas in patients who suffer from sporadic CRC a large fraction of ACF were found to be hyperplastic with a lower potential for malignant progression [2]. In humans most sporadic colorectal tumors are located in the middle part of the colon and in the rectum [7]. This specific location of tumors is also found after DMH-treatment of rodents [8]. DMH has been shown to cause DNA damage by alkylating DNA, resulting in the pro-mutagenic lesion *O*^6^-methylguanine (*O*^6^-MeG) [9], that is known to induce GC→AT transitions [10]. Such mutations are typically found in various genes linked to colon cancer, like *β-catenin *[11] or *K-ras *[12].

In humans IGF-II was suggested to play a role in development of CRC, as substantiated by findings showing that IGF-II is overexpressed in about 44% of all colorectal tumors due to a loss of imprinting (LOI) in the *IGF2 *gene [13]. The LOI characterizes a cancer-prone state since it is specifically found in the transformed colonic mucosa of most patients with CRC, but occurs rarely in individuals without cancer [13].

On a molecular basis, the importance of IGF-II for stimulation of growth was shown in several colon cancer cell lines [14,15]. Moreover, there is evidence, that IGF-II interferes with wnt-signaling by inducing the redistribution of β-catenin from the plasma membrane to the nucleus, as was shown in rat bladder carcinoma cells [16]. Although it has not been demonstrated so far that IGF-II affects β-catenin localization also in CRC, the development of CRC is almost invariably associated with nuclear accumulation of β-catenin [17], leading to complexes with the transcriptional repressors of the TCF/LEF family and the activation of target genes encoding growth promoting factors [18].

Besides the observation that IGF-II exhibits strong mitogenic potential, prevention of apoptosis could be another mechanism through which IGF-II might enforce tumor development [19]. Interference with the wnt-signaling pathway could play a role in this regard in so far as activation of this pathway was demonstrated to reduce the protein levels of caspase-3, 7 and 9 [20].

Finally, IGF-II might enhance the spread of CRC by the induction of lymphangiogenesis, as this process contributes to the infiltration of metastases in lymph nodes [21] and has been shown to be increased *in vivo *in the cornea of mice through IGF-II [22].

In the present study, we used IGF-II transgenic mice in order to investigate the influence of IGF-II on the development of ACF, adenomas and carcinomas in the colon that were induced by the chemical carcinogen DMH. Immunohistochemical staining of 5-bromo-2'-deoxyuridine (BrdU) and cleaved caspase-3 served to assess the effects of IGF-II on proliferation and apoptosis rates *in vivo*, respectively. Moreover, staining of β-catenin in the tumors served to indicate an activation of the wnt-signaling cascade. Quantitative detection of LYVE-1, a marker for lymphangiogenesis, was used to reveal effects of IGF-II on metastasis. In addition to IGF-II, serum levels of IGF-I and IGF-binding protein-2 (IGFBP-2) were assessed in order to exclude that decreases in IGF-I and increases in IGFBP-2 antagonize the IGF-II effects in a compensatory manner [23].

## Materials and methods

### Identification of PEPCK-IGF-II transgenic animals and carcinogen treatment

Female NMRI mice, overexpressing human IGF-II under the control of the rat phosphoenolpyruvate carboxykinase (PEPCK) promoter in several tissues including colon [24] and their non-transgenic female NMRI littermates were used in this study. To distinguish transgenic mice and their non-transgenic littermates animals were genotyped by PCR using the following primers for IGF-II: 5' ATG GGA ATC CCA ATG GGG AAG 3' and 5' CTT GCC CAC GGG GTA TCT GGG 3'. These primers generate a 336 bp product from the transgenic human *IGF2 *DNA [25]. Genomic DNA was prepared from mouse-tail biopsies. Samples were digested with Kawasaki buffer and ProteinaseK. PCR products were analyzed by agarose gel electrophoresis with ethidium bromide under UV light. The PCR-analysis revealed that 60 of the 124 animals were transgenic.

To investigate the impact of IGF-II on different stages of colon cancer development, IGF-II transgenic and non-transgenic mice were allocated into two groups, respectively, treated either with DMH or not (controls). DMH was applied by intraperitoneal (i.p.) injections of 40 mg/kg body weight DMH (Sigma, Deisenhofen, Germany) as aqueous solution [0.9% NaCl, 1 mM EDTA] once a week for 6 weeks from the age of 4 weeks on (group1: IGF-II-transgenic, early stage n = 24, late stage n = 7; and group3: wild-type, early stage n = 25, late stage n = 10). Transgenic and non-transgenic control animals received saline [0.9% NaCl, 1 mM EDTA] instead of DMH (group2: IGF-II transgenic, early stage n = 22, late stage n = 7, and group4: wild-type, early stage n = 24, late stage n = 5). All mice had free access to tap water and a standard diet (V1534; Ssniff, Soest, Germany), and were weighed weekly and monitored closely for signs of illness. None of the groups showed significant differences in weight gain. All experiments were carried out according to the German Animal Protection Law (2112531-37/00).

### RT-PCR

Tissue samples from the colons of PEPCK-IGF-II transgenic mice and non-transgenic littermates were immediately frozen on dry ice and stored at -80°C. Tissues were homogenized in Tri-Pure™ isolation reagent (Roche Diagnostics, Mannheim, Germany) using an ULTRA-TURRAX System T25 (ART, Mühlheim, Germany), and total RNA was prepared according to the manufacturer's instructions. 2.5 μg RNA were reversely transcribed into cDNA for 60 min at 37°C using 1× first strand buffer (Tris/HCl, pH8.3, KCl, MgCl_2_) (Invitrogen, Karlsruhe, Germany), 10 mM DTT (Invitrogen), dNTPs (1 mM each), 25 ng/μl Oligo(dT) primer and 20 U M-MLV reverse transcriptase (Invitrogen). The reaction was terminated for 5 min at 95°C. The amplification of human and murine IGF-II sequences was performed with species-specific primers as described [25]. Both primer-pairs yielded an amplification product of 499 bp [25]. The integrity of cDNA was confirmed by β-actin specific primers 5' GGC ATC GTG ATG GAC TCC G 3' (β-actin: forward), 5' GTC GGA AGG TGG ACA GCG A 3' (β-actin: reverse). PCR analyses were carried out in 20-μl reactions containing 2 μl cDNA, 0.5 U Taq polymerase (Qiagen), 50 μM dNTPs, 1× PCR-buffer [Tris-HCl, KCl, (NH_4_)_2_SO_4_, 1.5 mM MgCl_2_, pH 8.7 at 20°C], 1× Q-Solution (Qiagen) and 0.1 μM of both sense and antisense primers. β-actin reactions contained additional 1.5 mM MgCl_2 _(Qiagen). The amplification was performed as follows: samples were heated at 94°C for 4 min followed by 40 cycles of 94°C for 1 min, 60°C for 1 min and 72°C for 2 min. After a final extension at 72°C for 10 min samples were analyzed by agarose gel electrophoresis.

### Determination of serum IGF-II, IGF-I and IGFBP-2

Blood samples were taken by orbital puncture from mice under ether anesthesia before cervical dislocation. Samples were stored at 4°C for 30 min and centrifuged twice for 10 min at 10000 × *g *at 4°C to obtain serum. Serum samples were stored at -20°C until further analysis. Serum concentrations of IGF-II, IGF-I and IGFBP-2 were determined by radioimmunoassays (RIAs), which were standardized in the hormone laboratory of the Children's Hospital, University of Tübingen as previously described [24].

### Aberrant colonic crypt analysis and evaluation of tumor development

Animals were killed by cervical dislocation 5 (early stage) or 34 (late stage) weeks after the last carcinogen or saline injection, respectively. The colons were removed and rinsed with ice-cold Tris buffer (pH7.4). The colons were dissected along the longitudinal median axis, then placed on microscopic slides with the mucosal side up and fixed flat between filter paper and the microscopic slide in 10% neutral buffered formalin for three hours. The colonic crypts were stained with 2 g/l of methylene blue in PBS for 10–15 min. The number of ACF and the aberrant crypt multiplicity were determined by light microscopy at 25-fold magnification. All colons were scored by one observer. Several groups were rescored blindly. A few colons were repeated by a second observer and an inter-observer variation of less than 3% was observed. At the late stage, visible tumors were numbered and their location was recorded. The colon was fixed and stained for ACF and microadenomas as described before. Thereafter, each specimen was examined under a light microscope. Tumors were cut out and fixed in 10% neutral buffered formalin for further 20 hours. All tumors in the colon were routinely processed and embedded in paraffin. Before embedding, the tumors were measured in three perpendicular directions using a slide calliper. The gross tumor volume was calculated using the equation V = 4/3 π r^3^, where r was the average tumor radius. The tumors were cut in the middle and embedded with the section flat down. Serial tissue sections (3–4 μm) were made and mounted on glass slides.

### Histopathological grading of tumors

Hematoxylin and eosin (H&E)-stained serial sections were used for histological grading. The grading of tumors was based on pleomorphic morphology and invasive growth of the tumor cells, penetrating the lamina muscularis mucosae. The sections were analyzed by light microscopy at 400 times magnification.

### Immunohistochemistry (IHC)

For determination of the proliferative activity in the neoplasms, two hours (h) before sacrificing, mice were injected i.p. with 30 mg/kg body weight BrdU. BrdU is a pyrimidine analog which is incorporated by DNA-synthesizing nuclei and used for the identification of S-phase cells and was found to give the most accurate reflection of the proliferation rate [26]. Tissue samples were obtained and processed as described above. Paraffin-embedded serial tumor sections were routinely dewaxed, rehydrated and digested for 2 min with 0.01% trypsin before endogenous peroxidase was quenched with 3% H_2_O_2 _in Tris-HCl buffer pH7.8. After blocking non-specific protein-protein interactions with 1% BSA in Tris-HCl buffer for 60 min at room temperature, the slides were incubated with mouse-anti-BrdU monoclonal antibody (Sigma) diluted 1:10 in blocking buffer for 90 min at room temperature. Subsequently, secondary peroxidase-conjugated antibody directed to IgG from mouse (Sigma) was diluted 1:10 in Tris-HCl buffer containing 1% BSA and incubated 60 min at room temperature. Slides were thoroughly washed with Tris-HCl buffer between the incubation steps. All sections were counterstained with hematoxylin and mounted in entellan (Merck). Brown nuclei indicated cells in S-phase.

For determination of apoptosis in the colon tumors, IHC for cleaved caspase-3 was performed on formalin-fixed and paraffin-embedded tissue samples. Sections were dewaxed in xylene, endogenous peroxidase was blocked with 0.5% H_2_O_2 _in methanol and epitope retrieval was performed by microwaving for 33 min in citrate buffer (pH6.0) in a microwave pressure cooker at 750 W. The rabbit polyclonal cleaved caspase-3 (Asp175) antibody (Cell Signaling, Frankfurt, Germany) was diluted 1:200 in blocking buffer (5% goat serum in TBS, pH7.6) and incubated overnight. Biotinylated anti-rabbit IgG (dilution 1:200) (Vector Laboratories, Wertheim, Germany) served as secondary antibody. The Avidin Biotin Complex Vectastain *Elite *Peroxidase-based system (Vector Laboratories) with diaminobenzidine as the substrate (Sigma) served for visualization of cleaved caspase-3. Sections were counterstained as described for BrdU staining. Brown nuclei indicated cells that underwent apoptosis.

To demonstrate the presence and localization of β-catenin in the colorectal tumors, slides were pretreated as described for cleaved caspase-3 staining and incubated with a β-catenin-specific antibody (clone14, BD Pharmingen, Heidelberg, Germany) with strong reactivity against mouse β-catenin in a dilution of 1:10000. Biotinylated anti-mouse IgG (dilution 1:200) (Vector Laboratories) served as secondary antibody. Visualization of β-catenin and counterstaining was done as described for cleaved caspase-3 staining. Positive staining for β-catenin was detected in the nuclei, the cytoplasm and the cell membranes.

For visualization of lymphatic vessels in the tumors, treatment of slides and incubation steps were performed as described for cleaved caspase-3. For IHC rabbit polyclonal LYVE-1 antibody (DCS, Hamburg, Germany) was used in a dilution of 1:50. Brown cell membranes indicated positive staining.

Negative controls for all stainings were done by omitting primary antibodies. No positive staining was obtained in the case of cleaved caspase-3 and LYVE-1 staining. There was a slight signal in the negative control of β-catenin staining with the biotinylated anti mouse IgG of the tubular lumen and of immune cells in the stroma, but not of epithelial cells.

### Evaluation of BrdU and activated caspase-3 labeling

Tumors were scanned microscopically (×50 and ×100) to identify areas of most intense (hot spots) nuclear staining. The percentage of immunoreactive tumor cell nuclei of either BrdU or activated caspase-3 positive cells (proliferative rate, apoptosis labelling index) were calculated by counting 900–1500 epithelial cells at magnification (×200) within selected areas of at least five representative microscopic fields. Images were captured with the microscope coupled to a camera and digitalized on a computer. All brown nuclei and non-stained nuclei (blue) were marked before the percentage of positive staining was calculated. All tumors were scored by two observers, with one person blind to the different groups. Scored regions were chosen by the individual observer. The coefficient of variation between the two observers was in general lower than 5%.

### Evaluation of β-catenin localization

Staining intensity of the cell membrane was compared with non-tumorous epithelial cells that served as an internal positive control, and was graded into three categories: loss, reduced and preserved. Loss = disappearance of membrane staining in more than 80% of the tumor cells. The reduced type was split into cases with weak (loss in about 10–20%), moderate (loss in about 20–50%), and strong (loss in more than 50% of the cells) reduction of membrane staining. In the preserved type, tumor cells were found to be homogeneously positive for membrane staining. The staining of the epithelial tumor cells was evaluated under light microscopy by one observer and confirmed by a second one independently.

Nuclear staining of positive cells was defined as intense brown color in the nucleus. The pattern of nuclear immunohistochemical staining was evaluated under the light microscope by one observer and confirmed by a second one independently. The scored adenomas were classified as follows: negative or scattered (no or very few scattered positive cells without any clusters), focal positive cells clustered in focal areas (+ = positive cells in some small areas ~15–20%, ++ = positive cells in wide parts of the tumor ~20–25%, +++ = positive cells in most of the tubular constructions ~25–30%), diffuse (positive cells distributed diffusely and found in the vast majority of tubular constructions of the tumor >30%).

Immunohistochemical staining intensity of the cytoplasm was evaluated by microscopic images of immunostained sections and quantified based on optical density using an image analysis system (Optimas Software, Media Cybernetics, Silver Spring). Cytoplasmic intensity of tumor tissue (T) was analyzed by randomly processing at least 100 tumor epithelial cells in areas which were previously scored under light microscope for loss of, strongly reduced, weakly reduced, moderately reduced and preserved membrane staining, respectively, at ×400 magnification in 4 different microscopic fields of each region. As an internal control for each section, normal cytoplasmic intensity (N) was measured in 100 normal epithelial cells from the colon crypts. The definition of the percentage of cytoplasmic intensity was as follows: (1-N/T) × 100.

### Analysis of the lymphatic network

The overall lymphatic vessel density (number of lymphatic vessels per square millimeter) and lymphatic vessel area (area of lymphatic vessels per square μm) were determined. The occurrence of lymphatic vessels in the tumor tissue and in the stalk was assessed. The origin of the stalk arises from the stroma of the normal colon and spreads into the tumor, therefore this tissue builds a connection between normal colon stroma and the reactive stroma of the tumor. Image analysis was done using computer software. In brief, images from the whole tumor tissue were captured with the microscope coupled to a camera and digitalized on the computer. Lymphatic vessels were counted and the areas of vessels were automatically calculated by the software, after manually surrounding the vessel.

### Statistical analysis

Data were expressed as the mean ± SE, and were analyzed using Graph Pad Prism Version 3.0 (Prism; Graph Pad, San Diego, CA). Analysis of normally distributed data was performed using Student's t-test. Non-parametric data were analyzed using Mann-Whitney U-test. Differences were considered as statistically significant at *P *< 0.05.

## Results

### Expression of human IGF-II in the colon of transgenic mice

The expression of human IGF-II mRNA in the colon was analyzed by RT-PCR and could only be detected in PEPCK-IGF-II transgenic mice. Treatment with DMH did not induce the expression of endogenous IGF-II at the mRNA-level, neither in transgenic animals nor in their non-transgenic littermates with β-actin used as an expression control (data not shown).

### Serum IGF-II, IGF-I, and IGFBP-2 levels

**Figure 1:**
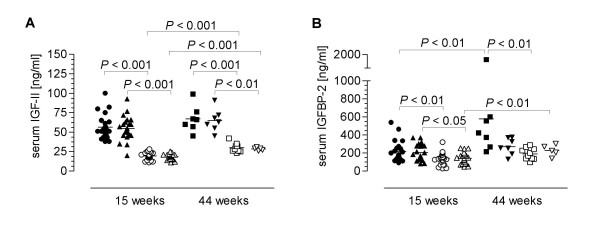
Effect of DMH-treatment and age on the serum level of IGF-II (**A**) and IGFBP-2 (**B**) in PEPCK-IGF-II transgenic (tg) and wild-type (wt) mice. IGF-II and IGFBP-2 were measured 11 and 40 weeks after the first DMH-application or in untreated age-matched controls by RIA. ●, ■ tg + DMH; ▲, ▼ tg without DMH; ○, □ wt + DMH; △, ▽ wt without DMH.

DMH-treatment did not influence the serum IGF-II concentrations neither in the transgenic mice nor in the non-transgenic mice and neither at 11 weeks (15 weeks old mice) nor at 40 weeks (44 weeks old mice) after the first DMH application (Fig. 1A). IGF-II-levels in non-transgenic mice were significantly lower than in transgenic mice at both time points measured (Fig. 1A). In the wild-type mice there was a slight but significant increase of IGF-II serum levels over time (19.52 ± 0.92 ng/ml versus 29.8 ± 1.9 ng/ml in DMH-treated, 18.72 ± 0.68 ng/ml versus 28.4 ± 0.87 ng/ml in non-treated animals at the age of 15 and 44 weeks, respectively) that was not observed in IGF-II transgenic animals (Fig. 1A).

Serum IGF-I concentrations were not affected by DMH-treatment or genotype nor did they change over time (data not shown).

DMH-treatment also had no influence on serum IGFBP-2 levels in both genotypes and at both time points measured (Fig. 1B). Although not as prominent as observed for IGF-II levels, IGFBP-2 concentrations in the serum of 15 week old transgenic mice were significantly higher than in the serum of wild-type mice (Fig. 1B) and correlated positively with IGF-II levels (*P *< 0.05, r^2 ^= 0.18; data not shown). At 44 weeks serum IGFBP-2 levels were significantly increased in transgenic mice treated with DMH versus DMH-treated wild-type mice but no longer different between both genotypes when untreated (Fig. 1B).

### Development of ACF

**Figure 2:**
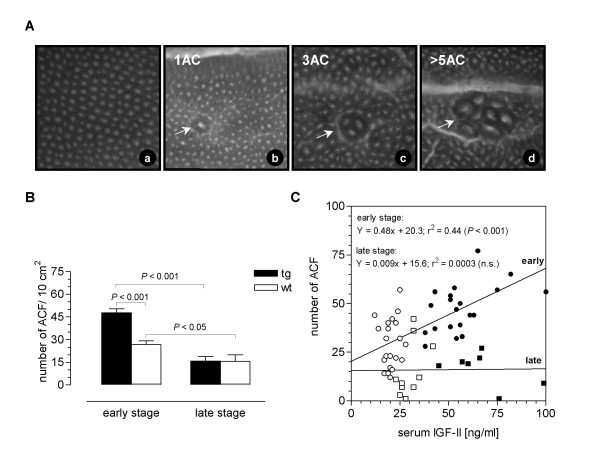
ACF-development in IGF-II transgenic (tg) and non-transgenic (wt) mice. **(A) **Photographs of methylene blue-stained mucosa of colons from animals treated with vehicle (**a**) or with six i.p. injections of DMH (**b, c, d**). Photographs were made at the age of 15 or 44 weeks. Shown is an example of normal colon mucosa and examples of ACF with different numbers of AC (objective 5 × 0.5). (**B**) ACF in DMH-treated mice in the early (11 weeks after first DMH-treatment) and late (40 weeks after first DMH-treatment) stages of tumor development. (**C**) Correlation between serum IGF-II and number of ACF per mouse in DMH-treated PEPCK-IGF-II transgenic (●, ■) and wild-type mice (○, □).

Single aberrant crypts (AC) and clusters of AC, aberrant crypt foci (ACF) consisting of multiple AC (Fig. 2A), were found in IGF-II transgenic as well as in non-transgenic mice after DMH-treatment, but not when treated with vehicle only. The total number of ACF was significantly higher (*P *< 0.001) in the colons of IGF-II transgenic mice (47.66 ± 2.62 per 10 cm^2^) than in wild-type mice (26.68 ± 2.48 per 10 cm^2^) at 11 weeks after the first DMH application (Fig. 2B). This difference in ACF development was no longer present after additional 29 weeks (IGF-II transgenic: 15.67 ± 3.12 per 10 cm^2 ^versus wild-type: 15.56 ± 4.45 per 10 cm^2^) (Fig. 2B). In the late stage of carcinogenesis at 40 weeks after the first DMH application, especially in the IGF-II transgenic mice the occurrence of the ACF was found to be drastically reduced (*P *< 0.001) versus the earlier stage (Fig. 2B). Since serum IGF-II levels in transgenic animals were still higher at this time point when compared to wild-type mice, IGF-II seems not to affect numbers of ACF at a later stage (Fig. 2B). In contrast, in the earlier stage of carcinogenesis a clear correlation between total numbers of ACF and serum IGF-II was found (Fig. 2C). Although IGF-II increased all clusters of ACF at five weeks post DMH-treatment, especially those consisting of ≥ three AC were enhanced (Table 1). The number of ACF with ≥ three AC remained constant over time, whereas the reduction in total number of ACF in both genotypes at a late stage was due to the reduced number in ACF containing one to three AC (Table 1).

**Table 1:** Numbers of ACF with different crypt multiplicity in IGF-II transgenic (tg) and wild-type (wt) mice at different time points

		**number of ACF**		
		
AC/focus	genotype	early stage	late stage	*P*-value
***1 AC***	tg	12.4 ± 1.0^a^	2.6 ± 1.2	*P *< 0.001
	wt	8.7 ± 0.9	1.7 ± 0.7	*P *< 0.001
**2 AC**	tg	17.4 ± 1.0^a^	6.0 ± 1.4	*P *< 0.001
	wt	12.9 ± 1.4	6.0 ± 1.7	*P *< 0.01
***3 AC***	tg	12.7 ± 1.0^c^	4.1 ± 0.8	*P *< 0.001
	wt	5.9 ± 4.6	4.6 ± 1.4	
***4 AC***	tg	4.2 ± 0.7^c^	2.3 ± 0.6	
	wt	1.1 ± 0.2	0.9 ± 0.5	
≥***5 AC***	tg	1.5 ± 0.3^a^	1.9 ± 0.6	
	wt	0.7 ± 0.3	2.6 ± 1.1	*P *= 0.071

^a^*P *< 0.05,^c^*P *< 0.001 significance of differences between the two genetic groups. The *P*-value in the last row expresses the significance of difference between different time points within the same genotype.

### Tumor development

**Figure 3:**
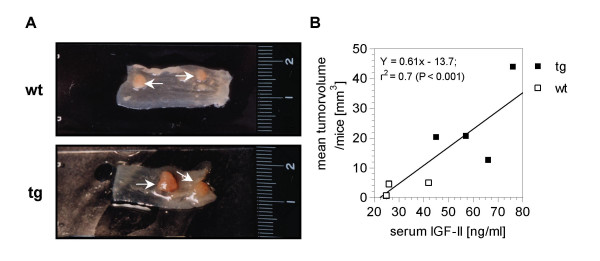
Effects of IGF-II on tumor volume. (**A**) Tumors in the colon (arrows) of a wild-type (wt) (upper panel) and an IGF-II transgenic (tg) animal (lower panel). (**B**) Significant positive correlation between IGF-II-level in serum and the mean tumor volume.

Following DMH-treatment, 4/7 (57%) PEPCK-IGF-II transgenic mice and 4/10 (40%) non-transgenic littermates developed tumors within 34 weeks. The transgenic animals developed 12 tumors in total, whereas in wild-type mice 8 tumors were found. The vast majority of the tumors found in the colon of wild-type mice were located in the distal colon (7/8) and only one tumor was located in the mid-colon. In contrast, IGF-II transgenic mice bore 50% of tumors (6/12) in the mid-colon. In both genotypes, no tumors were found in the proximal colon. Importantly, the tumor incidence and the tumor number per animal were not affected by IGF-II. However, an increased tumor size was observed in IGF-II transgenic mice (Fig. 3A) with a mean tumor volume (21.4 ± 5.7 mm^3^) 5.1-fold higher (*P *= 0.012) than in wild-type mice (4.2 ± 1.0 mm^3^). The tumor volume was clearly positively correlated with IGF-II serum levels (Fig. 3B).

### Histopathological and histochemical analysis

All 20 tumors found in the colon of DMH-treated mice were evaluated histologically. The histological analysis of the tumors revealed that the DMH injected mice mainly developed adenomas (non-transgenics: 5/8; IGF-II-transgenics: 11/12). Two of the colon tumors in wild-type mice and one colon tumor of an IGF-II transgenic mouse were classified as adenocarcinomas. One malignant lesion in wild-type mice was classified as an adenosquamous carcinoma, based on malignant squamous epithelial cells. The classification of carcinoma was based on pleomorphism and invasiveness into the lamina muscularis mucosae and into blood vessels.

### Proliferation and apoptotic indices of tumor epithelium

**Figure 4:**
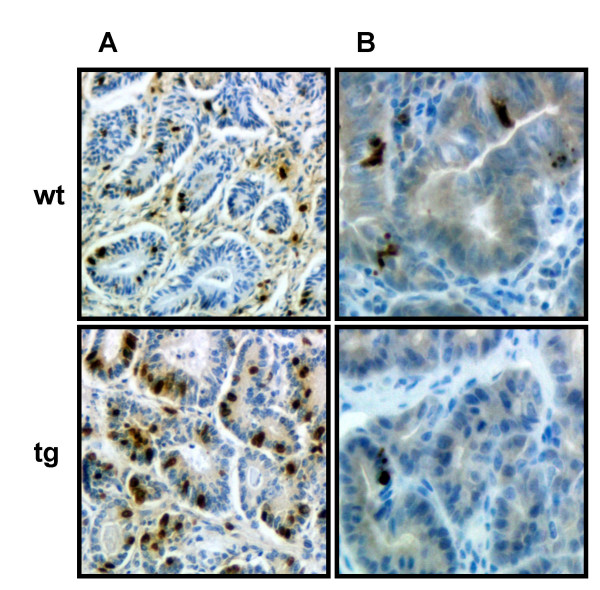
Nuclear immunohistochemical staining of proliferation and apoptosis markers in adenomas from IGF-II transgenic (tg) or wild-type (wt) mice. (**A**) Determination of S-Phase cells with BrdU (objective 10 × 1). (**B**) Determination of apoptotic epithelial cells in the tumor by an anti-cleaved caspase-3 antibody (objective 20 × 1).

To assess whether IGF-II affects epithelial cell proliferation or apoptosis in the neoplasms of the colon, BrdU-incorporation in S-phase cells and positive staining for cleaved caspase-3 was visualized in all tumors. Examples for the anti-BrdU staining in adenomas from transgenic and non-transgenic mice are shown in Figure 4A and examples of nuclear immunoreactivity versus cleaved caspase-3 in apoptotic cells of adenomas from transgenic and wild-type mice are given in Figure 4B.

The fraction of BrdU positive cells in adenomas of IGF-II transgenic mice (24.86 ± 1.57%) was much higher than in wild-type mice (16.84 ± 4.56%) (*P *< 0.05).

The relative presence of nuclei stained for cleaved caspase-3 was generally very low in all tumors of both genotypes. Wild-type mice showed more apoptotic epithelial cells (1.69 ± 0.54%) in the adenomas than IGF-II transgenic animals (0.94 ± 0.28%) without reaching statistical significance.

### Distribution of β-catenin staining in adenomas

**Figure 5:**
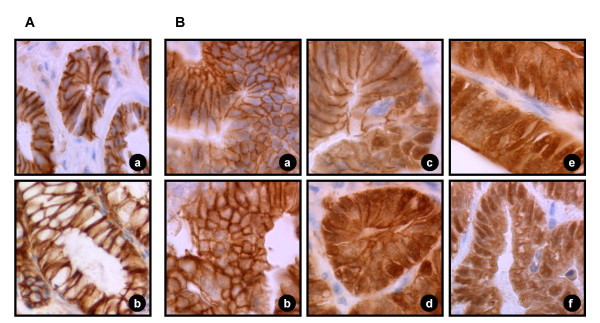
Immunohistochemical staining of β-catenin in adenomas of DMH-treated animals (objective 40 × 1). (**A**) non tumoric colon tissue (**a**) and normal colon crypts (**b**) show a strong positive staining for β-catenin in the membrane but not in the cytoplasma or in the nucleus. (**B**) Adenomas with preserved membrane, no nuclear and low cytoplasmic (**a**) or weak cytoplasmic (**b**) staining of β-catenin. Reduced (weak) membrane, weak cytoplasmic and focal (+) nuclear staining is shown in (**c**). Reduced (moderate) membrane, strong cytoplasmic and focal (++) nuclear staining of β-catenin is given in (**d**). Loss of membrane staining with strong staining of the cytoplasma and focal (+++) nuclear staining of β-catenin is shown in (**e**) and loss of membrane staining with moderate staining of the cytoplasma and diffuse distribution of nuclear staining in the aberrant tissue of the tumor in (**f**).

The results of cellular β-catenin localization in adenomas of wild-type and IGF-II transgenic animals are shown in Tables 2, 3 and 4 and examples of different patterns of distribution are shown in Figure 5.

**Table 2:** Cell membrane expression of β-catenin in adenomas from IGF-II transgenic (tg) and wild-type (wt) mice

	***preserved***	***reduced***			***loss***
	
genotype		weak	moderate	strong	
tg	1 (11.1%)	1 (11.1%)	3 (33.3%)	2 (22.2%)	2 (22.2%)
wt	2 (40%)	1 (20%)	1 (20%)	1 (20%)	0 (0%)

*Preserved*: no or < 5% loss of membrane staining; *Reduced*: weak (loss in about 10–20%); moderate (loss in about 20–50%) and strong (loss in over 50%); *Loss*: loss of membrane staining was evident in more than 80%.

**Table 3:** Cytoplasmic expression of β-catenin in adenomas of IGF-II transgenic (tg) and wild-type (wt) animals

	**low**	**week**	**moderate**	**strong**
	
genotype	+	++	+++	++++
tg	0 (0%)	1(11.1%)	5 (55.6%)	3 (33.3%)
wt	1 (20%)	2 (40%)	2 (40%)	0 (0%)

Elevation of cytoplasmic staining compared to the internal non-tumor epithelial tissue. low: (+ = non to 5%); week: (++ = 5–15%); moderate: (+++ = 15–25%); strong: (++++ = over 25%)

**Table 4:** Nuclear expression of β-catenin in adenomas of IGF-II transgenic (tg) and wild-type (wt) mice

	**negative/scattered**	**focal**		**diffuse**	
	
genotype		+	++	+++	
tg	1 (11.1%)	2 (22.2%)	3 (33.3%)	2(22.2%)	1 (11.1%)
wt	2 (40%)	1 (20%)	1 (20%)	1(20%)	0 (0%)

Negative/scattered: no or < 5% scattered positive cells; Focal: positive cells clustered in focal areas (+ = 15–20%; ++ = 20–25%; +++ = 25–30%); Diffuse: positive cells distributed diffusely over the whole tumor.

In wild-type mice 60% of the adenomas exhibited a preserved (Fig. 5Ba,b, Table 2) or a weakly reduced (Fig. 5Bc, Table 2) staining of the cell membrane, whereas only 22.2% of the adenomas from the IGF-II transgenic mice revealed this type of staining (Table 2). Adenomas from IGF-II transgenic animals also displayed a higher fraction of moderately or strongly reduced membrane staining (Fig. 5Bd, Table 2). The almost complete loss of membrane staining was restricted to adenomas from IGF-II transgenic mice (Fig. 5Be,f, Table 2).

The reduction of cell membrane staining in adenomas from IGF-II transgenic mice was associated with an increased staining of β-catenin in the cytoplasm, e.g. 89% of the adenomas from IGF-II transgenic animals, but only 40% of the adenomas from wild-type mice showed a cytoplasmic staining intensity exceeding that of non-tumorous tissue by more than 15% (Fig. 5Bd–f, Table 3). Weak or low cytoplasmic staining was mainly found in the adenomas of wild-type mice, whereas only one adenoma of the IGF-II transgenic mice exhibited cytoplasmic staining lower than 15% (Fig. 5Ba–c, Table 3). The mean fractions of cytoplasmic staining intensity in adenomas from IGF-II transgenic (25.52 ± 2.39%) and wild-type mice (11.83 ± 3.4%) adenomas were significantly different (*P *< 0.01).

Simultaneous to the cytoplasmic increase in β-catenin staining nuclear staining intensities were found to be enhanced in adenomas from IGF-II transgenic mice. Diffuse presence of nuclear β-catenin staining was only found in an adenoma of an IGF-II transgenic mouse but not in adenomas of wild-type mice (Table 4), and was accompanied by a complete loss of membrane-specific staining (Fig. 5Bf). Focal nuclear staining was also found at higher rates in adenomas from IGF-II transgenic animals (Fig. 5Bc–e, Table 4). Moreover, absence of nuclear staining was found in 40% of adenomas from wild-type mice, but only in 11% of adenomas from IGF-II transgenic mice (Fig. 5Ba,b, Table 4).

### Lymphangiogenesis

**Figure 6:**
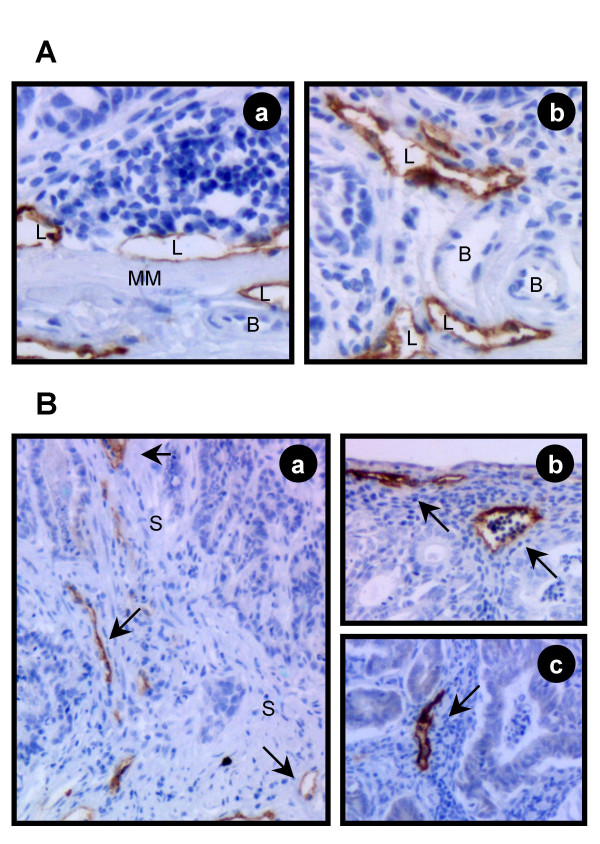
Immunohistochemical staining of lymphatic vessels by the LYVE-1 antibody in normal colon tissue and colon tumors. (**A**) LYVE-1 expression was restricted to thin walled lymphatic endothel (L), whereas blood vessels (B) remained unstained in the normal colon (**a**) as well as in tumor tissue (**b**). Immunohistochemical staining (objective 20 × 1). Lymphatic vessels are scattered along the normal colon and penetrate the muscularis mucosae (MM). (**B**) In pedunculated adenomas Lyve-1 positive vessels (arrows) were found in the tumor stalk (S) (**a**), in the mid (**b**) and marginal (**c**) parts of the tumor. Immunohistochemical staining (objective 10 × 1).

Staining of lymphatic endothelial cells was clear and distinct, whereas endothelial cells in blood vessels were not stained in both normal and tumorous colon (Fig. 6A). Moreover, lymphatic endothelium was localized in connective tissues such as submucosa and lamina propria (Fig. 6A, left panel). In pedunculated adenomas the lymphatic endothelium pervaded the tumor tissue through the stalk, which penetrated the tumor (Fig. 6Ba), and was also found in the connective tissue in the middle (Fig. 6Bb) and marginal parts of the tumors (Fig. 6Bc). The LVD (lymphatic vessel density) and the proportion area of lymphatic vessels (mean area of endothelial vessels per area tumor tissue) of endothelium in the tumors of both genotypes were approximately 2.5 per mm^2 ^and 1%, respectively. Also the distribution of the lymphatic endothelium within the tumors did not differ between the two genotypes.

## Discussion

IGF-II is suggested to play a role in the development and progression of colorectal tumors [23,27]. On a molecular basis, cell culture studies provided insights into the mode of action by which IGF-II might influence processes, which are relevant for carcinogenesis [14,15]. Moreover, animal studies showed that IGF-II plays a role for cancer development also *in vivo *[28,29]. Evidence for a crucial role of IGF-II for colorectal cancer development in humans stems from a report demonstrating overexpression of IGF-II in tumorous colonic tissue, as a consequence of loss of imprinting in the *IGF2 *gene [13,30]. Effects of IGF-II overexpression on the development of colorectal cancer have been investigated previously in the Apc^Min/+ ^mouse, containing a loss-of-function mutation in the *Apc *gene [29]. This mutation is found in familial adenomatous polyposis coli [31], but also during sporadic colon cancer development in humans [32]. Although Apc^Min/+ ^mice are used as a model for the investigation of colorectal tumor development it has to be stressed that Apc^Min/+^mice develop tumors predominantly in the small intestine, questioning whether they really represent a valid model for sporadic colon cancer development in humans. To circumvent these obvious obstacles, we initiated colorectal cancer using the chemical carcinogen DMH, that induces tumors specifically in the descending colon of rodents [7,8]. The relevance of the present model is further shown by the histopathological findings in DMH-treated mice, that were similar to those observed in human sporadic colon tumors [33]. Using IGF-II transgenic mice we tested how IGF-II affects early and late stages of chemically induced colorectal cancer *in vivo*.

### DMH-treatment does not affect IGF-II expression

IGF-II transgenic mice displayed almost three-fold higher serum IGF-II levels than non-transgenic mice at 11 weeks after the first DMH application, and more than two-fold higher serum IGF-II levels at 34 weeks after complete DMH initiation. Serum IGF-II levels were independent of DMH-application. However, since the liver carcinogen diethylnitrosamine has been shown to induce re-expression of endogenous IGF-II in the liver of mice in a dose-dependent fashion [25], we tested in addition whether DMH influences the *IGF2 *mRNA expression in the colon. DMH treatment did not induce the expression of *IGF2 *in the colon of mice, indicating that the effect of IGF-II on preneoplastic alterations (ACF) in the colon was due to the IGF-II transgene.

### IGF-II increases the number of ACF and the tumor size but not the tumor incidence

Non-treated IGF-II transgenic mice did not develop ACF, whereas IGF-II transgenic mice treated with DMH exhibited significantly more ACF in an early phase of tumor development than the non-transgenic DMH-treated mice. These data demonstrate that the development of ACF requires an initiating stimulus. Moreover, it shows that IGF-II alone is not able to initiate tumor development but it increases precancerous lesions once initiation has been triggered. Interestingly, at an early stage of tumor development IGF-II transgenic mice displayed predominantly more ACF containing ≥three AC. In the later stages in both genotypes, but more prominent in IGF-II transgenic mice, the ACF with less than three AC declined. Since the number of tumors did not differ significantly between transgenic and non-transgenic animals it is suggested that the number of ACF present at a later stage of carcinogenesis is more important for tumor development than the number at an early stage or the number of AC within an ACF. In this regard, IGF-II seems to provide a growth-advantage at an early stage that can be eliminated perhaps due to repair mechanisms. When an ACF at a later stage progresses towards a tumor, however, IGF-II again provides a growth advantage as is evidenced by the increased tumor volumes in IGF-II transgenic mice. Since IGF-II is a positive regulator for proliferation in colon cancer cells [15], it is possible that overexpression of IGF-II may lead to a temporarily higher development of hyperplastic or atypical crypts, which are well detectable by methylene blue staining in the early stage of colon cancer development, but with a higher potential to regress [6]. That overexpression of IGF-II in this model acts as a promotor and not as an initiator of tumor development has to be concluded from similar tumor numbers in IGF-II transgenic and wild-type mice. This observation is supported by findings in colorectal cancer cell lines, that IGF-II was not able to stimulate the transformation of cells [15]. A slight but insignificant increase in colorectal cancer incidence with increased IGF-II levels, as observed in our study, was also shown to be present in humans [23,34].

### The mitogenic effect of IGF-II is associated with increased accumulation of β-catenin in the cytoplasm and nucleus

It is widely accepted that IGFs promote mitogenic effects [35,36]. In accordance with this, we found that adenomas in IGF-II transgenic mice have significantly more cells in the S-phase than adenomas of wild-type mice. The mitogen-activated protein (MAP) kinase and β-catenin pathways have been shown to promote proliferation in different cancer cells when activated [37,38] and several members of both pathways are known to be hotspots for genetic alterations commonly found in colon cancer [39-41]. In tumors MAP kinases can either be activated by IGF-II via the IGF-1R [42] or via the insulin receptor-A (IR-A) [35]. As a matter of fact, by immunohistochemical staining of activated MAP kinase42/44 we found a strong expression in the aberrant epithelia of colon tumors but not in normal colon tissue, whereas there was no visible difference of staining in tumors from IGF-II transgenic and wild-type animals (data not shown).

Staining for β-catenin revealed an increased accumulation mainly in the cytoplasm, and to a lower extent in the nuclei of adenomas from IGF-II transgenic compared to adenomas from wild-type mice with a concomitant decline of β-catenin staining at the plasma membrane. Such stabilized levels of cytoplasmic β-catenin should pass into the nucleus to increase transcriptional activity [43,44]. A similar redistribution of β-catenin from the plasma membrane to the cytoplasm and nucleus was also found in epithelium to mesenchyme transition (EMT) [16] that characterizes in embryonic development the process of conversion of epithelial cells to mesenchymal cells [45], but is also a characteristic of cancer [46].

Besides the mitogenic actions, IGFs are discussed to be antiapoptotic [47]. Moreover, antiapoptotic effects have been shown to be triggered by *wnt-1 *signaling through IGFs [48]. Therefore, we evaluated whether IGF-II affects apoptosis rates in colon tumors as well. In tumors of IGF-II transgenic and wild-type mice only low rates of apoptosis could be detected which is in accordance with the impaired ability of tumors to control cell mass [49]. However, IGF-II did not show a significant effect on apoptosis suggesting that activation of the β-catenin pathway in adenomas of mice has no considerable impact on apoptosis rates.

### Activation of the β-catenin-pathway is associated with increased IGFBP-2 serum levels in transgenic animals

IGFBP-2 possesses moderately preventing effects on the development of colorectal cancer [50] most likely by antagonizing the effect of serum IGF-II [23]. On the other hand in the case of a manifested colorectal cancer, serum IGFBP-2 levels are significantly elevated and correlate with the stage of disease [51]. Since *IGFBP2 *is a target gene of the β-catenin/TCF/LEF complex [52] it was not astonishing that we observed significantly higher IGFBP-2 serum levels in IGF-II transgenic mice and a positive correlation between serum IGF-II and IGFBP-2. Given that the increase of IGF-II in transgenic animals was much more pronounced than the increase of IGFBP-2 it could be concluded that IGFBP-2 is not able to block completely the effects of the transgene.

The higher IGFBP-2 levels in DMH-treated transgenic animals at the early and late stage of tumor development were not associated with any effect on tumor incidence. It has to be stressed here, however, that this is the case under simultaneously increased IGF-II levels and may be different when IGF-II is less increased. That the biological effects of IGF-II are strictly dependent on IGFBP-2 can be further derived from the increase of both IGF-II and IGFBP-2 in wild-type mice with age. In the progressed stage of tumor development IGFBP-2 seems not to be able to promote any longer growth inhibiting activities [51] but enhanced expression might represent an effort to antagonize the effects of growth factors. In accordance with this, in our studies one animal showed tremendously increased IGFBP-2 levels at a later stage that correlated well with its very poor status because of development of several large adenomas spreading over the middle and distal colon.

### Lymphangiogenesis in colon tumors is not affected by IGF-II overexpression

The importance of lymphangiogenesis for the malignant spread of colorectal tumors has been previously reported [53]. Moreover, it was described that IGF-II is able to induce lymphangiogenesis in the mouse cornea *in vivo *[22]. In accordance with a study showing LYVE-1, a specific lymphatic endothelial marker, to be located in the submucosa of the small intestine [54] we detected LYVE-1 positive endothelia also in the submucosa of the normal colon. In addition, in adenomas and adenocarcinomas of the colon we detected LYVE-1 positive lymphatic vessels reaching the tumor through the stromal stalk and penetrating through the connective tissue (lamina propria) into the tumor, a characteristic not described so far. Nevertheless, there was no effect of IGF-II on lymphangiogenesis in tumors investigated.

### Summary

In conclusion, IGF-II is a growth promoter during early and late stages of colorectal cancer development *in vivo*. It enhances the growth of initiated cells leading to ACF with higher crypt multiplicity and also of tumor cells leading to higher tumor volumes. These effects of IGF-II were associated with increased cytoplasmic and nuclear accumulation of β-catenin. IGF-II, however, *in vivo *seems not to be involved in the transformation process as becomes evident by the lack of effects on chemically induced number of tumors.

## Abbreviations

AC: aberrant crypts

ACF: aberrant crypt foci

BrdU: 5-Bromo-2'-deoxyuridine

CRC: colorectal cancer

DMH: 1,2 dimethylhydrazine

IGF-II: insulin-like growth factor II

PEPCK: phosphoenolpyruvate carboxykinase

## Competing interests

The author(s) declare that they have no competing interests.

## Authors' contributions

DD is the principle investigator of this study, who carried out the bulk of experiments, summarized the data and drafted this manuscript. In the Lab of MWE the serum IGF-II and IGFBP-2 RIAs were standardized. AH critically read the manuscript. HL, EW (department chair) and DO proposed the study design and assisted in writing the manuscript. All authors read and approved the final manuscript
